# The Treated Drug Users in the Slovak Republic

**Published:** 2017-09

**Authors:** Pavol BENO, Martin SAMOHYL

**Affiliations:** 1. Dept. of Laboratory Medicine in Health Care, Faculty of Health Sciences and Social Work, Trnava University, Trnava, Slovak Republic; 2. Institute of Hygiene, Faculty of Medicine, Comenius University, Bratislava, Slovak Republic

## Dear Editor-in-Chief

Drug craving and the other compulsive behaviours are the essences of addiction. People who abuse substances are likely to find themselves increasingly isolated from their families. Several drug abuse studies have been conducted (1–3).

The aim of this study was to analyse of treated drug users in the Slovak Republic in the period 2006–2015. We conducted a descriptive analysis the treatments for drug abuse were obtained from the National Health Information Centre of the Slovak Republic (4).

Data were transformed to relative percentage annual difference and averaged, thus we are so called mean percentage difference (MPD) characteristic of annual growth consumption of medicaments, formally

$$\mathrm{MPD}=\frac{1}{n}\sum \frac{x_{i}-x_{i-1}}{x_{i-1}}*100$$

Where ***x*** represents input vector of patients treated drug abuse with period ***n*** in years ***i***.

In 2015, 2720 patients underwent treatment for the use of illegal drugs in the Slovak Republic (84% males; 50.1 persons treated per 100000 inhabitants) (4).

In the period 2006–2015, the highest increase of MPD was in treated users in the category other drugs (males: MPD 20.1%; females MPD 27.4%) with a significant decrease for male users of injectable substances (MPD −6.1%) and female users of heroin (MPD −4.6%) (Table 1). The highest MPD was observed in males being treated for drug use in the age group 40–44 yr (23.0%) and in women in the age group 45–49 yr (MPD 15.6%) (Table 2).

**Table 1:** Treated drug users according to the type of drug and gender per 100000 inhabitants, 2006–2015

**Year**	**Gender**	**Type of Drugs**					
		**Heroin**	**Other types of drugs**	**Cocaine**	**Stimulants**	**Injectable substances**	**Cannabis**
**2006**	Male	22.1	1.8	0.7	12.9	3.6	10.4
	Female	6.3	0.5	0.1	3.5	0.5	1.4
**2007**	Male	20.2	4.2	0.3	14.5	3.6	11.2
	Female	5.9	0.8	0.1	3.6	0.3	1.0
**2008**	Male	21.6	3.5	0.3	17.3	3.0	11.0
	Female	6.5	0.5	0.1	3.3	0.5	0.7
**2009**	Male	15.8	5.6	0.3	15.9	2.7	11.5
	Female	4.6	1.8	0.1	4.1	0.2	0.9
**2010**	Male	18	6	0.5	23.1	2.1	14.4
	Female	4.3	1.8	0.1	4.8	0.4	0.9
**2011**	Male	16.1	4.8	0.5	24.2	2.5	13.8
	Female	4.1	1.0	0.1	5.4	0.3	1.1
**2012**	Male	12.2	3.3	0.4	28.0	2.0	15.5
	Female	3.8	0.6	0.0	5.7	0.2	0.9
**2013**	Male	12.9	4.6	0.4	29.8	2.0	19.5
	Female	2.7	0.8	0.1	7.1	0.3	1.6
**2014**	Male	11.3	4.5	0.8	32.7	1.9	17.6
	Female	3.5	1.0	0.0	7.2	0.4	1.5
**2015**	Male	13.1	5.0	0.5	35.8	1.9	21.3
	Female	3.6	0.9	0.2	6.9	0.2	1.9
**MPD^1^**		−4.5	20.1	5.8	12.8	−6.1	9.0
**MPD^2^**		−4.6	27.4	-*	8.4	4.6	8.0

1 mean percentage difference in males

2 mean percentage difference in females

* In 2012 and 2014, no females were treated for cocaine addiction because we were not able to evaluate MPD

**Table 2:** Treated drug users according to their age groups and gender per 100000 inhabitants, 2006–2015

**Year**	**Gender**	***Age groups (yr)***									
		**0–14**	**15–19**	**20–24**	**25–29**	**30–34**	**35–39**	**40–44**	**45–49**	**50–54**	**≥ 55**
**2006**	Male	6.0	141.7	197.6	173.8	85.9	45.8	11.0	8.1	3.6	1.2
	Female	2.3	45.1	55.1	37.4	18.1	16.1	8.9	5.6	3.9	1.3
**2007**	Male	4.5	135.3	208.4	183.3	104.0	45.8	16.9	6.7	5.6	1.5
	Female	1.0	36.2	52.1	39	22.9	13.1	10.1	6.2	3.4	1.5
**2008**	Male	3.9	137.2	221	202.9	117.9	42.0	18.2	8.9	5.1	1.1
	Female	-	35	43.4	42.5	23.8	11.1	8.1	4.7	3.4	1.9
**2009**	Male	3.0	125.7	202.9	170.2	115.7	44.4	21.3	7.4	4.1	0.9
	Female	0.2	31.1	48.1	42.6	27.8	8.1	8.8	5.8	5.4	1.7
**2010**	Male	3.1	136.2	250	208	147.8	68.7	25.2	10.0	5.7	1.2
	Female	1.5	40.4	39.9	43.1	35.4	11.6	8.4	3.1	5.0	2.2
**2011**	Male	2.8	135	232.8	234.2	158.4	79.3	27.6	9.6	7.4	3.6
	Female	0.5	40.3	43.8	48.8	26	18.5	9.0	8.0	7.2	2.0
**2012**	Male	2.8	143.2	230.8	225.6	145.4	74.8	26.0	10.3	9.1	2.3
	Female	1.5	39.2	47.8	39.3	23.9	14.8	9.4	3.8	4.7	1.8
**2013**	Male	2.8	148	249.6	260.5	175.2	95.0	43.8	17.6	12	2.3
	Female	1.5	54.3	44.1	37.5	28.4	16.8	12.3	3.9	3.7	2.6
**2014**	Male	3.5	116.2	239.6	248.7	193.1	113.5	44.9	15.1	8.7	3.0
	Female	2.5	44.9	43.8	52.9	30.7	20.7	16.0	7.9	2.7	2.7
**2015**	Male	2.3	158.5	273.1	268.5	206.8	132.7	60.7	19.1	12.0	2.6
	Female	1.0	42.6	46.1	52.0	33.3	22.9	12.9	6.8	6.5	2.1
**MPD^1^**		−8.6	2.3	4.1	5.6	10.8	13.9	23.0	13.4	17.9	21.6
**MPD^2^**		-*	1.0	−1.4	4.8	8.4	7.7	5.7	17.8	15.6	7.5

1 mean percentage difference in males

2 mean percentage difference in females

* was not possible to evaluate due to a lack of data for women in the year 2008
